# Alcohol use disorders among people living with HIV/AIDS in Southern Brazil: prevalence, risk factors and biological markers outcomes

**DOI:** 10.1186/s12879-017-2374-0

**Published:** 2017-04-11

**Authors:** Cláudio Moss da Silva, Raúl Andrés Mendoza-Sassi, Luisa Dias da Mota, Maíba Mikhael Nader, Ana Maria Barral de Martinez

**Affiliations:** Faculty of Medicine, Federal University of Rio Grande – FURG, Rio Grande, RS Brazil

**Keywords:** HIV/Aids, Alcohol induced disorders, Prevalence, Risk factors, Immunologic markers, Brazil

## Abstract

**Background:**

Alcohol abuse is an important public health problem, frequently unrecognized among people living with HIV/AIDS (PLWHA), and requires investigation and intervention. It is usually associated with lower adherence to highly active antiretroviral therapy (HAART). It can also produce adverse clinical outcomes, such as changes in certain HIV markers, particularly CD4 cell counts and HIV viral loads (VLs). Thus, this study aimed to evaluate the prevalence of alcohol abuse among PLWHA, its associated risk factors and effects on CD4 cell counts and HIV VLs in southern Brazil.

**Methods:**

Between December 2012 and July 2013, 343 patients were interviewed at a reference hospital in southern Brazil. The instrument used was the Alcohol Use Disorder Identification Test (AUDIT), and a cutoff of eight points or more was applied. Socioeconomic, demographic, clinical and laboratory data were also collected. The statistical analysis included a Poisson regression to evaluate the factors associated with alcohol use disorder, and a linear regression was performed to assess the relationship between AUDIT scores and CD4 cell counts and HIV VLs.

**Results:**

Alcohol abuse was present in 28.6% of the respondents, and possible dependence was present in 5%. The risk factors identified included being male, mixed or black skin color, low education and the use of intravenous or inhaled drugs. A higher AUDIT score was associated with a lower CD4 cell count but was not associated with higher HIV VL values.

**Conclusions:**

Our results show the importance of screening for alcohol abuse in this group. The prevalence of alcohol abuse was high, and it was associated with socioeconomic factors and the use of illicit drugs. Moreover, AUDIT score negatively affected CD4 cell counts as well.

## Background

Alcohol abuse has been recognized as an important public health problem, common and frequently undiagnosed among people living with HIV/AIDS (PLWHA) and is amenable of intervention [1, 2]. Several studies have examined the prevalence of alcohol abuse and its associated factors among PLWHA. The data available are the result of research conducted within very different populations and regions, using different methodologies. Alcohol use disorders among PLWHA seems to be 2–4 times higher than those disorders in the general population [3–5], and it is estimated that 40 to 50% of these individuals have a history of alcohol abuse or dependence [1, 6].

This association can generate a higher disease burden for this group of individuals [7–12]. Among the negative effects related to high alcohol consumption is its ability to interfere with the immune system [13–15], predisposing the individual to the increased occurrence of bacterial infections, particularly tuberculosis [16, 17], enhancing the liver damage caused by co-infection with Hepatitis C Virus [18] and altering the metabolism of antiretroviral drugs [19, 20]. Alcohol consumption is also associated with harmful behavior such as smoking, illicit drug use and the adoption of unsafe sexual practices [21]. Although not always conclusive, several studies have suggested that alcohol consumption is also related to a lower adherence to highly active antiretroviral therapy (HAART) with consequent inadequate viral suppression, the potential emergence of viral resistance and treatment failure [22–24].

Certain factors affecting alcohol abuse and the consequences that such behaviors can have on the evolution of the disease are amenable to intervention. The identification of these factors may be useful in the implementation of actions aimed at controlling alcohol use and providing relevant information for detecting individuals at a higher risk of adverse clinical outcomes.

Another aspect of interest is the effect alcohol can have on certain clinical and laboratory parameters, which may be important in the evaluation of PLWHA and disease progression. Studies suggest that in these individuals, alcohol consumption may be associated with lower CD4 cell counts; however, this finding is not consistent in the literature [23, 25–33].

Therefore, the aim of this study was to analyze, in a sample of PLWHA in southern Brazil, the prevalence of alcohol abuse using the Alcohol Use Disorders Identification Test (AUDIT), to determine the risk factors associated with this alcohol abuse, and finally to study the independent effect of alcohol use on CD4 cell counts and HIV VLs.

## Methods

### Study design and population

This was a cross-sectional study conducted between December 2012 and July 2013 in the University Hospital of the Federal University of Rio Grande (Universidade Federal do Rio Grande – FURG), Rio Grande, RS, Brazil; this is a reference hospital for the care of HIV-infected patients in the southernmost part of the country.

### Participants and sample power

The participants were selected consecutively as they received outpatient care at the hospital’s HIV/AIDS service. They had documented HIV infection according to Brazilian Ministry of Health’s guidelines [34], were aged 18 or older and were capable of understanding and answering the proposed questionnaire. Participation in the study was voluntary, and there was no financial compensation. Of the 360 eligible individuals, 343 (95.3%) answered the proposed questionnaire and were included in this study. This sample size was selected to have a statistical power of 80% in detecting a difference of 100 cells/mm^3^ between the mean CD4 cell counts of the patients without (mean 600, standard deviation [SD] 300) and with (mean 500, SD 300) alcohol abuse, with a 95% confidence level and an unexposed to exposed ratio of 2.5:1.

### Instruments and variables

A structured pre-coded questionnaire was applied to all of the participants, and a review of their medical records was performed. Information regarding sociodemographic variables (gender, age, skin color, education, relationship status and per capita family income), lifestyle and habits (HIV risk behaviors, alcohol and illicit drugs consumption and number of sexual partners) were obtained from the interview. Medical history (length of HIV diagnosis, use of HAART, prior hepatitis and sexually transmitted diseases [STD] in the last year) and laboratory data (nadir and current CD4 cell counts and current HIV VL) were collected from the medical records. (Table 1.)

**Table 1:** Characteristics of the evaluated patients (*n* = 343)

Characteristics	n/ $$ \overset{-}{x} $$		(%)/SD
Male gender	174		(50.7)
Age (years)			
18–34	99		(28.9)
35–54	113		(32.9)
55–75	131		(38.2)
Skin color			
White	238		(69.4)
Mixed	55		(16.0)
Black	50		(14.6)
Education (years)			
< 8	185		(53.9)
≥ 8	158		(46.1)
Monthly family income per capita (quartiles) (US$)			
1st	111.0	±	31.9
2nd	201.5	±	31.5
3rd	325.0	±	27.6
4th	712.4	±	384.2
Homosexual/bisexual males	47		(13.7)
Married/Cohabiting	171		(49.9)
Sexual partners in the last year (n)			
0	62		(18.1)
1	188		(54.8)
2	38		(11.1)
≥ 3	55		(16.0)
STD previous year	42		(12.2)
AUDIT Zones			
I	245		(71.4)
II	71		(20.7)
III	10		(2.9)
IV	17		(5.0)
Use of intravenous drugs	46		(13.4)
Use of inhaled illicit drugs	111		(32.4)
Previous hepatitis	68		(19.8)
Using HAART	306		(89.2)
Never used HAART	28		(8.2)
Undetectable HIV VL	247		(72.0)
Current CD4 (cells/mm^3^)			
≥ 500	182		(53.0)
200–499	134		(39.1)
< 200	27		(7.9)

The AUDIT questionnaire was chosen to assess the alcohol use in the last year [35]. The scores obtained were grouped into four predefined risk zones applying the recommended cutoff points: Zone I - low risk use (0–7 points), Zone II - hazardous use (8–15 points), Zone III - harmful use (16–19 points) and Zone IV - likely dependence (20–40 points). Alcohol abuse is defined with an AUDIT score of eight or more points [35]. The questionnaire has good sensitivity and specificity in the detection of alcohol abuse [35]; moreover, it has been used to evaluate PLWHA [25, 36–38] and was previously validated for use with the Brazilian population [39].

CD4 cell counts were determined by flow cytometry using FACSCalibur (Becton Dickinson Immunocytometry Systems, Oxford, United Kingdom) operating with TruCOUNT tubes; the results are expressed as a continuous variable in cells/mm^3^. HIV VLs were determined by polymerase chain reaction using Abbott Real Time HIV-1 (Abbott Laboratories, Abbott Park, IL, USA); the results are presented as base 10 logarithms as a continuous or categorized variable (detectable or undetectable). The minimum detection value for HIV VL was 40 copies/ml (Log_10_ 1.60). Current CD4 cell counts and HIV VLs measurements were performed within the year of the survey (mean 3 months).

All data were entered into and reviewed in a database created for that purpose. The data were then exported to Stata 13.1 (Statacorp LP, College Station, TX, USA).

### Statistical procedures

#### Description of the sample

Sample characteristics were presented as the mean ± SD, or median and percentile if not normally distributed, for continuous variables and as proportions for categorical variables. The prevalence of alcohol abuse and its 95% confidence interval (95CI) were also calculated.

#### Analysis of risk factors associated with alcohol abuse

The crude prevalence ratios (PRs) were calculated along with their respective 95CIs, and *p* values were calculated using the Wald test. A Poisson regression was used for the multivariate analysis with robust calculation of variances and using a backward stepwise analysis. All of the variables were entered following a 3-level hierarchical model [40]. The first level contained the sociodemographic variables, the second level contained the habits and customs variables, and the third level contained the clinical variables. All variables with a *p* value of less than or equal to 0.20 were maintained for fitting with the next level to consider the possibility of a negative confounder. The significance test used was the Wald test. In the case of ordinal variables, the Wald test was used to evaluate linear trends. When there was no ranking, the Wald test was used to assess heterogeneity.

#### Effect of alcohol use on the CD4 cell counts and HIV VL values

Multiple linear regression was used to evaluate the independent effect of alcohol use (measured as AUDIT score), on dependent variables (CD4 cell counts and HIV VL values). A backward stepwise regression was performed and the AUDIT score was adjusted to confounders associated with alcohol use. All variables were included in the model, but only variables with a *p* value ≤0.05 were maintained. The F-test was used to assess the *p* value of AUDIT score. Residuals histogram and diagnostic plot of residuals vs. fitted values was used to verify the normal distribution of the regression residuals.

In all statistical tests a *p*-value of ≤0.05 for a 2-tailed test was used.

## Results

### Description of the sample

The male/female ratio was 1.03, the mean age was 42.2 years (SD 11.1) with a range of 18 to 75 years, and there was a predominance of individuals with white skin color. The participants had a median of 7 years of education (P25 = 5, P75 = 11), 54% had not finished elementary school. The median per capita monthly income was US$ 254.25 (P25 = 155.50, P75 = 375.00) and the income of the upper quartile was six times greater than that of the lower quartile. The median time of HIV diagnosis was 7.9 years (P25 = 3.7, P75 = 11.1), the median nadir CD4 cell count was 155 cells/mm^3^ (P25 = 54, P75 = 272) and the median current CD4 cell count was 540 cells/mm^3^ (P25 = 345, P75 = 765). The median AUDIT score was four points (P25 = 1, P75 = 8). Forty-six patients (13.4%) did not drink alcohol, 98 (28.6%) have alcohol abuse (score ≥ 8 points) and 17 (5.0%) showed possible dependence (score ≥ 20 points). Other characteristics of the study patients are shown in Table 1.

### Analysis of risk factors associated with alcohol abuse

Table 2 shows the PR values after adjusting for the studied factors. At the 1st level (sociodemographic), the following variables remained as risk factors: male gender (PR 2.88, 95CI 1.96–4.24), mixed skin color (PR 1.10, 95CI 0.74–1.65), black skin color (PR 1.44, 95CI 0.98–2.13) and 2nd income quartile (PR 1.67, 95CI 1.08–2.60). Protective factors included years of education (PR 0.95, 95CI 0.90–0.99) and being in the 4th income quartile (PR 0.67, 95CI 0.37–1.21). Among the behavioral factors (2nd level), intravenous drug use (PR 1.45, 95CI 1.04–2.02) and the use of inhaled illicit drugs (PR 1.85, 95CI 1.28–2.68) were identified as risk factors. At the 3rd level, neither the length of HIV infection diagnosis nor the use of HAART, were significant in either the crude or the adjusted analysis.

**Table 2:** Multivariate analysis of factors associated with alcohol abuse among HIV positive patients (*n* = 343)

Variable	n (%)	Crude PR (95CI)	p	Adjusted PR (95CI)	p
Gender^c,f^					
Female	27 (16.0)	1	0.001	1	0.001
Male	71 (40.8)	2.55 (1.73–3.77)		2.88 (1.96–4.24)	
Age^c,f^					
18–34	27 (27.3)	1	0.5^b^	1	0.08^a^
35–54	59 (30.7)	1.13 (0.76–1.66)		0.81 (0.56–1.16)	
55 or older	12 (23.1)	0.85 (0.47–1.53)		0.61 (0.34–1.10)	
Skin color^c,f^					
White	60 (25.2)	1	0.01^a^	1	0.04^a^
Mixed	17 (30.9)	1.23 (0.80–1.93)		1.10 (0.74–1.65)	
Black	21 (42.0)	1.67 (1.12–2.47)		1.44 (0.98–2.13)	
Education^c,f^					
Each year		0.93 (0.89–0.98)	0.005	0.95 (0.90–0.99)	0.03
Income (quartiles)^c,f^					
1st	22 (23.7)	1	0.006^b^	1	0.003^b^
2nd	33 (40.2)	1.70 (1.08–2.67)		1.67 (1.08–2.60)	
3rd	29 (34.1)	1.44 (0.90–2.31)		1.18 (0.75–1.88)	
4th	14 (16.9)	0.71 (0.39–1.30)		0.67 (0.37–1.21)	
Married/Cohabiting^c^					
No	53 (30.8)	1	0.3	1	0.7
Yes	45 (26.3)	0.85 (0.61–1.20)		0.94 (0.69–1.29)	
STD^d^					
No	80 (26.6)	1	0.02	1	0.5
Yes	18 (42.9)	1.61 (1.08–2.40)		1.15 (0.79–1.68)	
Number sexual partners^d^					
Up to 2	76 (26.4)	1	0.03	1	0.2
3 or more	22 (40.0)	1.52 (1.04–2.21)		1.25 (0.90–1.74)	
Use of intravenous drugs^d,f^					
No	70 (23.6)	1	0.001	1	0.03
Yes	28 (60.9)	2.58 (1.89–3.52)		1.45 (1.04–2.02)	
Use of inhaled illicit drugs^d,f^					
No	43 (18.5)	1	0.001	1	0.001
Yes	55 (49.5)	2.67 (1.92–3.71)		1.85 (1.28–2.68)	
Previous hepatitis^d^					
No	71 (25.8)	1	0.02	1	0.5
Yes	27 (39.7)	1.54 (1.08–2.19)		1.14 (0.80–1.60)	
Length of HIV infection^e^					
Each year		0.997 (0.965–1.031)	0.9	0.989 (0.957–1.021)	0.5
Use of HAART^e^					
No	7 (18.9)	1	0.2	1	0.2
Yes	91 (29.7)	1.57 (0.79–3.13)		1.69 (0.81–3.54)	

^a^Linear trend test; ^b^Heterogeneity test; ^c^1^st^ level; ^d^2^nd^ level; ^e^3^rd^ level; ^f^final model

### Effect of alcohol use on the CD4 cell counts and HIV VL values

Table 3 shows the effect of the AUDIT score on the CD4 cell count and HIV VL adjusted to other explanatory variables. An association was found between the AUDIT score and the number of CD4 cells, with a regression coefficient of −5.14 (*p* = 0.04). Therefore it was observed a drop of five cells/mm^3^ for every one point increase in the AUDIT score, even when adjusted for other factors. No change was observed for HIV VLs measurements. Residuals histogram and diagnostic plot of residuals vs. fitted values showed a normal distribution.

**Table 3:** Linear regression coefficients of AUDIT score for CD4 cell counts and viral load values (*n* = 343)

	Crude				Adjusted^b^			
	Coeff.^a^	95CI	P	R^2^	Coeff.	95CI	P	R^2^
CD4 cells	−9.18	−13.90/−4.46	0.001	0.038	- 5.14	−10.23/−0.04	0.04	0.078
(cells /mm^3^)								
Viral load	0.005	−0.01/0.02	0.6	0.002	0.01	−0.005/0.03	0.1	0.33
(Log_10_ HIV RNA)								

^a^Coefficient, ^b^Adjusted for age, gender, skin color, education, income, relationship status, STD, number of sexual partners, previous hepatitis, length of HIV infection, use of HAART and use of intravenous or inhaled drugs

## Discussion

This study found a high prevalence of alcohol abuse among PWLHA that exceeded the values found in the general population. The most important factors associated with alcohol abuse were male gender, mixed or black skin and the use of illicit drugs (inhaled and/or intravenous). In contrast, patients in the 4th income quartile and with more education had a reduced risk. The hypothesis that alcohol can alter laboratory parameters was confirmed by observing that the number of CD4 cells decreased linearly as the AUDIT risk zone increased. Supporting this hypothesis, the linear regression revealed that every increase of one unit on the AUDIT score reduced the CD4 cell count by more than five cells/mm^3^.

Studies show that alcohol consumption among PWLHA is frequent and may be higher than in the general population. In our study, the prevalence of alcohol abuse was more than three times higher than that previously observed in the same region for the general population [41]. In 2014, the United States of America (USA) reported a prevalence of alcohol use disorder of approximately 7% in the population aged 18 or older [42]; furthermore, several USA studies show high alcohol consumption among PLWHA, where 8–30% of subjects were classified as “heavy drinkers” [3, 27, 31, 43]. In other countries, the prevalence of alcohol abuse among PLWHA evaluated with the application of the AUDIT questionnaire showed similar or even much higher rates than those found here. These rates are affected by the habits and customs of the region in which each study is carried out [28, 37, 44]. The consistently higher prevalence of alcohol abuse among PLWHA that is observed in different geographic areas, cultures and socioeconomic conditions provides evidence that this behavior can be contextualized to the condition of seropositivity and possibly to behavioral aspects.

The probability of the outcome was nearly three times higher among males. This finding is consistent with biological, behavioral and social aspects determining that men drink more alcohol and are at a greater risk of abusing the substance [45]. Socioeconomic factors were also identified as important risk/protection factors for abuse; this outcome was more prevalent among individuals with mixed and black skin and less prevalent among individuals with a higher income and/or education. Other studies conducted both in Brazil and in other countries have also found that although the frequency of alcohol use is higher among more favored economic classes, alcohol abuse occurs more often among individuals of the less privileged social classes [46–49]. Regarding skin color, a gradient was observed in which the risk of alcohol abuse increased as the skin darkened. This fact can be explained by socio-economic aspects because there is a clear association in the population between skin color, economic class and education level [50]. This consistency of findings points to inequalities in health. Individuals with higher socioeconomic status and more education are more protected from alcohol abuse. This may be related to these individuals’ ability to better cope with problems and stressful situations due to their easier access to information and health services, which takes them to adopting a healthier lifestyle [51–53].

The factors associated with alcohol abuse in this study group are similar to those found in the general population [47], indicating that socioeconomic and demographic factors really are macro determinants of alcohol abuse and that the condition of seropositivity is part of a context that already predisposes an individual to this behavior.

We found that the AUDIT score can predict CD4 cell count, and therefore, individuals with high scores, may have very low cell counts. As the R^2^ of the model was low, other explanatory variables are influencing the variation of CD4 cell count. Regarding to VL, no association was found.

Studies evaluating the effects of alcohol consumption on these HIV markers have not shown consistent results. Among PLWHA not using HAART, some researchers have found an inverse association between alcohol consumption and CD4 cell counts [30, 31], whereas others have failed to show such results [26, 29, 33] or have only found it for heavy drinkers [27]. The same effect occurred when individuals receiving HAART were evaluated. Although some researchers have shown lower CD4 cell counts [25, 30, 31, 33, 54], others have found no significant differences [26, 27, 29, 55–58]. This inconsistency in findings therefore underscores the need for further studies on the subject. If we consider as valid the hypothesis that there is an inverse relationship between alcohol consumption and CD4 cell counts, the reasons for this phenomenon occurring appear to be multifactorial and related to immunological, toxic, nutritional and behavioral effects [59].

Studies evaluating the effect of alcohol consumption on HIV VL among individuals who were not receiving HAART found no association [27, 31, 33]; however, among individuals receiving HAART, higher VL values or even viral suppression failure have been demonstrated [2, 30, 31, 33, 56, 60]. This finding appears to be, at least in part, a reflection of lower adherence to HAART [2, 30, 33]. In the present study, no association was established between alcohol consumption and HIV VL, even when individuals who had undergone HAART for more than 6 months were selected.

This lack of consistency in the relationship between alcohol and HIV markers should be addressed with the implementation of new prospective studies. Using better definitions of the pattern and volume of alcohol consumed with control for cofactors, such as the length of HIV infection, the use of HAART and treatment adherence, type and amount of illicit drugs used, could facilitate a better understanding of this relationship. However, the findings of this study provide evidence that the use of the AUDIT score in clinical practice could be useful in predicting, at least in part, changes to the CD4 cell count in these individuals. Furthermore, the identification of alcohol abuse should prompt a better evaluation and counseling for abandoning or reducing this habit and consequently, prevent or minimize its adverse effects on the health of the individual.

### Study limitations

The limitations of this study include its cross-sectional design because this does not allow establishing a causal relationship, particularly between alcohol use and HIV markers. In the case of associated factors, in which the temporality between certain factors (such as age, gender and skin color) and the outcome appears to be maintained, reverse causality is less likely, despite the use of a cross-sectional design. Another limitation is the possible lack of statistical power for some of the studied associations. The AUDIT score was the alcohol exposure measure used to evaluate the effect on HIV markers, however the amount and frequency of consumption were not quantified. The positive result regarding CD4 cells cannot therefore be interpreted exclusively as a biological effect, but it should be considered that other mechanisms, such as behavioral mechanisms, may somehow also affect this marker. However, the effect remained even after adjusting for variables clearly related to the CD4 cell level, such as the disease duration, the use of HAART and the use of illicit drugs, which reinforces the association found. Finally, individuals who did not participate in the study could have affected the results because non-participation was related to non-attendance at the service and not to the refusal to participate. These individuals may have had lower adherence to HAART and may be major consumers of alcohol and illicit drugs, in which case the prevalence of alcohol abuse would have been underestimated and likewise the effect of risk factors. However, the percentage of losses did not exceed 5%, which minimizes the possibility of this selection bias.

## Conclusions

This study has shown that alcohol abuse among PLWHA is common and more frequent than expected in the general population. Alcohol abuse was associated with socioeconomic factors and the use of intravenous or inhaled illicit drugs. We have demonstrated here that AUDIT score negatively affected CD4 cell counts. These results highlights the importance of screening for alcohol abuse in this population.

## Abbreviations

95CI: 95% confidence interval
AIDS: Acquired immunodeficiency syndrome
AUDIT: Alcohol use disorder identification test
CD4: Cluster of differentiation 4
Coeff: Coefficient
FURG: Federal University of Rio Grande
HAART: Highly active antiretroviral therapy
HIV: Human immunodeficiency virus
P25: 25th percentile
P75: 75th percentile
PLWHA: People living with HIV/AIDS
PR: Prevalence ratio
SD: Standard deviation
STD: Sexualy transmited disease
USA: United States of America
VL: Viral load
Vs: Versus
WHO: World Health Organization
